# Supply chain management of laboratory commodities for tuberculosis in Indonesia: using assessment results to strengthen staff capacity

**DOI:** 10.1186/2052-3211-7-S1-P2

**Published:** 2014-12-17

**Authors:** Russ Vogel

**Affiliations:** 1 USAID|DELIVER PROJECT, John Snow Inc., Jakarta, Indonesia

## Background

High quality laboratory diagnosis is critical for any tuberculosis (TB) control program. Reliable and accurate laboratory testing depends on collecting high quality specimens, using careful collection methods, and properly storing and transporting specimens. Although various guidelines for proper collection and handling exist in Indonesia, there was no data on health worker compliance with those guidelines. Such data could help the Ministry of Health ensure staff capacity to carry out high quality laboratory diagnosis.

## Method

In September 2013, the USAID|DELIVER Project conducted an assessment of specimen handling from collection to storage, transport, and handling at receiving sites. The assessment also covered the availability of equipment for the storage, packaging, and use of personal protective equipment (PPEs); infectious waste handling; and the availability of standard operating procedures (SOPs). The National Tuberculosis Program (NTP) will use the results to strengthen the TB program and build staff capacity.

## Results

Packing of TB sputum specimens and transport to referral sites is ongoing in the Drug Resistant TB project and in other sentinel health centres in Indonesia. However, no uniform standard operating procedures (SOPs) exist or are enforced for the handling and transport of specimens. Most collectors are not using cold chain with thermometers in the shipment. Also, there are no measures to protect the community and the environment during transport of specimens, as required in United Nations/WHO regulations. Many collection sites and receiving laboratories need additional cold chain equipment, especially refrigerators, for specimen storage. Staff members need basic training.

## Discussion

Currently, there are no SOPs to protect the community and environment during transport of TB specimens in Indonesia. This includes the use of safe transport devices and labels for infectious material or hazardous substances, as required in UN/WHO regulations. Because the TB program will most likely be expanded to sites far from the current referral laboratories, more referral labs for cultures and drug susceptibility testing (DST) should be prepared and a more effective referral system developed. The MOH also needs to establish uniform SOPs for the way equipment/support devices are used by staff working on a variety of programs.

## Lessons learned

The MOH should prioritize improving the country’s ability to perform TB cultures, including staff capability to handle DST. SOPs should clearly state when, where and at what temperature the cold chain is needed for sputum and isolates. These new SOPs should be imparted to staff and enforced.

